# Editorial: The blue frontier: cancer research meets the diversity of marine chemistry and biology, new challenges, and prospects

**DOI:** 10.3389/fcell.2025.1540811

**Published:** 2025-02-06

**Authors:** Daniel L. Pouliquen, Susana P. Gaudêncio

**Affiliations:** ^1^ Inserm, CNRS, CRCI^2^NA, Nantes Université, Université d’Angers, Angers, France; ^2^ Associate Laboratory i4HB – Institute for Health and Bioeconomy, NOVA School of Science and Technology, NOVA University of Lisbon, Caparica, Portugal; ^3^ UCIBIO-Applied Molecular Biosciences Unit, Chemistry Department, Blue Biotechnology and Biomedicine Lab., NOVA School of Science and Technology, NOVA University of Lisbon, Caparica, Portugal

**Keywords:** marine natural products, anticancer activity, drug discovery, one health, blue biotechnology, secondary metabolites, polymers, enzymes

Marine ecosystems account for more than 80% of the planet’s biodiversity (Dayanidhi et al., 2021). Despite their immense potential for health, they have been studied far less than terrestrial ecosystems for the discovery of innovative drugs. Nonetheless, 15 marine-derived drugs have already been approved for commercialization (https://www.marinepharmacology.org/approved), including eight anticancer drugs. This vast marine biodiversity, along with the intricate and unique adaptations that enable survival in challenging physical environments, serves as a rich source of inspiration for scientists, driving innovative concepts and biotechnological developments. Over the past decades, several exciting areas of research have emerged and benefited from translational exchanges between marine sciences and oncology. Some examples include the “One World–One Health Concept” for the study of the impact of human activities on marine species oncogenesis, transmissible cancers, and tumor suppressor mechanisms (Dujon et al., 2021). The deep sea environment offers a vast reservoir of microorganisms, fungi, and invertebrates, complemented by algae and phytoplankton thriving in upper sea levels. Together, these organisms produce an extensive variety of primary and secondary metabolites, many of which exhibit potential anticancer and/or immunomodulatory properties (Molinski et al., 2009). Beyond small molecules, marine organisms also synthesize a diversity of macromolecules with unique biological, physicochemical, and structural properties, holding potential for by-product valorization, offering positive outcomes for both marine science and oncology (Claverie et al., 2020). The articles within this Research Topic explore various aspects of these compelling translational connections (https://www.frontiersin.org/research-topics/58861/the-blue-frontier-cancer-research-meets-the-diversity-of-marine-chemistry-and-biology-new-challenges-and-prospects/articles).

Aligned with the “One Health” principles, previous investigations on marine species populations affected by cancer identified transmissible cancers in bivalves (Yonemitsu et al., 2019) while providing insights into the pathogenesis of some common cancers between fishes and humans (Sarasamma et al., 2018). Based on multi-year, collaborative studies on marine mammal cancers (Gulland et al., 2020), an international team recently investigated fibropapillomatosis in green sea turtles (Whilde et al.). A survey conducted by 44 experts revealed that recent transcriptomic and genomic analyses of this disease shared many oncological molecular similarities with human cancers. This study also highlighted the efficacy of human anticancer medications and the need for more research combining histology and genomics.

Interest in deep sea microorganisms has grown significantly among the community of researchers working on anticancer natural products, building on the pioneering work of William Fenical’s laboratory (Marris, 2006). Advances in understanding the ecological roles of marine secondary metabolites have highlighted certain fungal species for their antagonistic properties against other microorganisms (Garo et al., 2003). Through a collaborative effort involving researchers worldwide, the list of potential antitumor marine products began to be enriched with compounds isolated from fungi (Mioso et al., 2017; Jimenez et al., 2020; Barreca et al., 2020). A new contribution to this field evaluated the cytotoxic activities of eremophilane-type sesquiterpenoids isolated from a species belonging to the *Emericellopsis* genus of Ascomycota fungi. Their investigation revealed a new molecule that presented interesting cytotoxic activities against five different human cancer cell lines (Virués-Segovia et al.).

By 2012, at least 88 novel alkaloids with cytotoxic activity on cancer cell lines, isolated from marine sponges, were documented (Mioso et al., 2017). Since then, this number continued to grow, including new discoveries originating from other sources such as bacteria, cyanobacteria, seaweed, fungi, ascidians, bryozoans, tunicates, or mollusks (Yu et al.). These metabolite structures have been divided into 20 distinct chemical classes, with six primary mechanisms of action described. Interestingly, many of these alkaloids are currently undergoing phase I/II clinical trials, offering promising prospects for future therapeutic applications.

The state and dynamics of water in biological systems have been the subject of considerable interest and many debates since the first quarter of the 20th century, with marine sciences playing an important historical role (McCutcheon and Lucké, 1926). As an increasing number of molecules isolated from oceanic sources exhibit interesting properties in oncology and/or immunology, integrating recent breakthroughs in biophysics, and molecular and cell biology represents an exciting challenge. The development of new concepts and ideas emerging from potential intellectual bridges between cancer research and marine biology may, therefore, pave the way for future advancements (Pouliquen).
